# The pluripotency transcription factor Nanog represses glutathione reductase gene expression in mouse embryonic stem cells

**DOI:** 10.1186/s13104-019-4411-0

**Published:** 2019-07-01

**Authors:** Claudia Solari, María Victoria Petrone, Ayelén Toro, Camila Vazquez Echegaray, María Soledad Cosentino, Ariel Waisman, Marcos Francia, Lino Barañao, Santiago Miriuka, Alejandra Guberman

**Affiliations:** 1 Departamento de Química Biológica/Laboratorio de Regulación Génica en Células Madre, Universidad de Buenos Aires, Facultad de Ciencias Exactas y Naturales, Buenos Aires, Argentina; 2 Instituto de Química Biológica (IQUIBICEN), CONICET - Universidad de Buenos Aires, Intendente Guiraldes 2160, Ciudad Universitaria, Pab. 2, 4to piso, QB-71, Buenos Aires, Argentina; 3 Laboratorio de Investigación de Aplicación a Neurociencias (LIAN), CONICET - Fundación para la Lucha contra las Enfermedades Neurológicas de la Infancia (FLENI), Buenos Aires, Argentina; 4 Departamento de Fisiología y Biología Molecular y Celular, Universidad de Buenos Aires, Facultad de Ciencias Exactas y Naturales, Buenos Aires, Argentina; 5 Consejo Nacional de Investigaciones Científicas y Técnicas (CONICET), Buenos Aires, Argentina

**Keywords:** Nanog, Glutathione reductase, Transcriptional regulation, Gene expression, Embryonic stem cells, Differentiation, Redox homeostasis

## Abstract

**Objective:**

Redox homeostasis maintenance is essential to bring about cellular functions. Particularly, embryonic stem cells (ESCs) have high fidelity mechanisms for DNA repair, high activity of different antioxidant enzymes and low levels of oxidative stress. Although the expression and activity of antioxidant enzymes are reduced throughout the differentiation, the knowledge about the transcriptional regulation of genes involved in defense against oxidative stress is yet restricted. Since glutathione is a central component of a complex system involved in preserving cellular redox status, we aimed to study whether the expression of the glutathione reductase (Gsr) gene, which encodes an essential enzyme for cellular redox homeostasis, is modulated by the transcription factors critical for self-renewal and pluripotency of ESCs.

**Results:**

We found that Gsr gene is expressed in ESCs during the pluripotent state and it was upregulated when these cells were induced to differentiate, concomitantly with Nanog decreased expression. Moreover, we found an increase in Gsr mRNA levels when Nanog was downregulated by a specific shRNA targeting this transcription factor in ESCs. Our results suggest that Nanog represses Gsr gene expression in ESCs, evidencing a role of this crucial pluripotency transcription factor in preservation of redox homeostasis in stem cells.

**Electronic supplementary material:**

The online version of this article (10.1186/s13104-019-4411-0) contains supplementary material, which is available to authorized users.

## Introduction

Maintaining the homeostasis of redox state is essential for cellular functions. A complex system composed by different enzymes and low molecular weight compounds, such as antioxidant vitamins and glutathione, is involved in preserving cellular redox status. Within this network, glutathione reductase (Gsr) is one of the most important enzymes, since it catalyzes the reduction of glutathione disulfide to the thiol form of glutathione (GSH), maintaining the pool of reduced GSH. This molecule is the most abundant non-protein thiol in cells and has an essential role as a cellular redox buffer. It is co-substrate of other antioxidant enzymes which break down the reactive oxygen species (ROS), generated as by-products of cellular respiration.

ROS play an important role as second messengers in various cellular functions such as proliferation, differentiation and apoptosis [1–5], hence their homeostasis is critical. However, an increase in the concentration of these species leads to an imbalance between oxidants and antioxidants, altering cellular redox homeostasis driving to oxidative stress. In this condition, ROS may be toxic due to their ability to modify macromolecules such as proteins, lipids and even damage DNA [6]. Such modifications could alter their biological function, thus impairing distinct cellular processes. Therefore, antioxidant compounds and enzymes are essential to maintain ROS at physiological levels necessary to mediate cellular responses and to minimize oxidative stress.

Embryonic stem cells (ESCs), which derive from the inner cell mass of blastocysts, possess several systems that secure genomic stability. This safeguard is essential since these cells physiologically originate all cell types of the organism, including the germ line. Thus, ESCs have high fidelity mechanisms involved in DNA repair, high activity of the multiple antioxidant enzymes and low levels of ROS with the consequent low mutation rate [7]. Furthermore, those cells that have accumulated mutations launch molecular mechanisms to undergo differentiation or apoptosis as an additional safeguard to preserve the stem cell genome [8]. Recent studies suggest that ROS have a role in the balance between self-renewal and differentiation in stem cells. In pluripotent stem cells the number of mitochondria is low and also their biogenesis rate. However, during differentiation, both ROS levels [7] and mitochondrial proliferation and activity increase [9], together with the ATP demand [7, 10]. Moreover, high levels of ROS promote differentiation of different types of stem cells [11–13]. On the other hand, ROS are necessary to maintain self-renewal in neural progenitors [5]. In addition, in ESCs induced to differentiate, GSH/GSSG ratio decreased as GSH is oxidized at the beginning of the differentiation protocol and then returned to similar levels respect to the undifferentiated state. Concomitantly, an augment in ascorbic acid levels occurred as a probable compensation to maintain homeostasis during ESC differentiation [14]. Regarding development, ROS are involved in hatching and may also be part of the regulatory system of programmed cell death in mouse blastocyst [6]. For these reasons, the increasing amounts of ROS results in a continuous decrease of glutathione levels challenging the antioxidant stress defense of the early embryo [15].

Some genes involved in the defense system against oxidative stress are modulated along ESCs differentiation [7, 10]. Based on the reported modulation of this system and its importance in securing genomic stability and cellular functions, we hypothesized that some of the antioxidant genes are regulated by the transcription factors fundamental for pluripotency, such as Oct4, Sox2 and Nanog. We have previously found that both *sod1* and *sod2* genes, that encode for superoxide dismutases, are induced by the pluripotency transcription factors, which are essential for ESCs’ self-renewal and pluripotency [16, 17].

Although it has been reported that expression and activity of antioxidant enzymes are reduced throughout the differentiation [1, 6], the knowledge about the transcriptional regulation of genes involved in defense against oxidative stress is yet restricted.

The aim of this work was to study whether the expression of the *Gsr* gene is modulated by the pluripotency transcription factors. We found that Glutathione reductase gene was expressed in undifferentiated ESCs and was upregulated when these cells were induced to differentiate, concomitantly with Nanog downregulation. In accordance with these results, we found that *Gsr* gene expression was induced in ESCs where Nanog gene was downregulated by transfection with shRNA vector targeting this transcription factor.

## Main text

### Results

#### Glutathione reductase gene is upregulated throughout differentiation

**Fig. 1:**
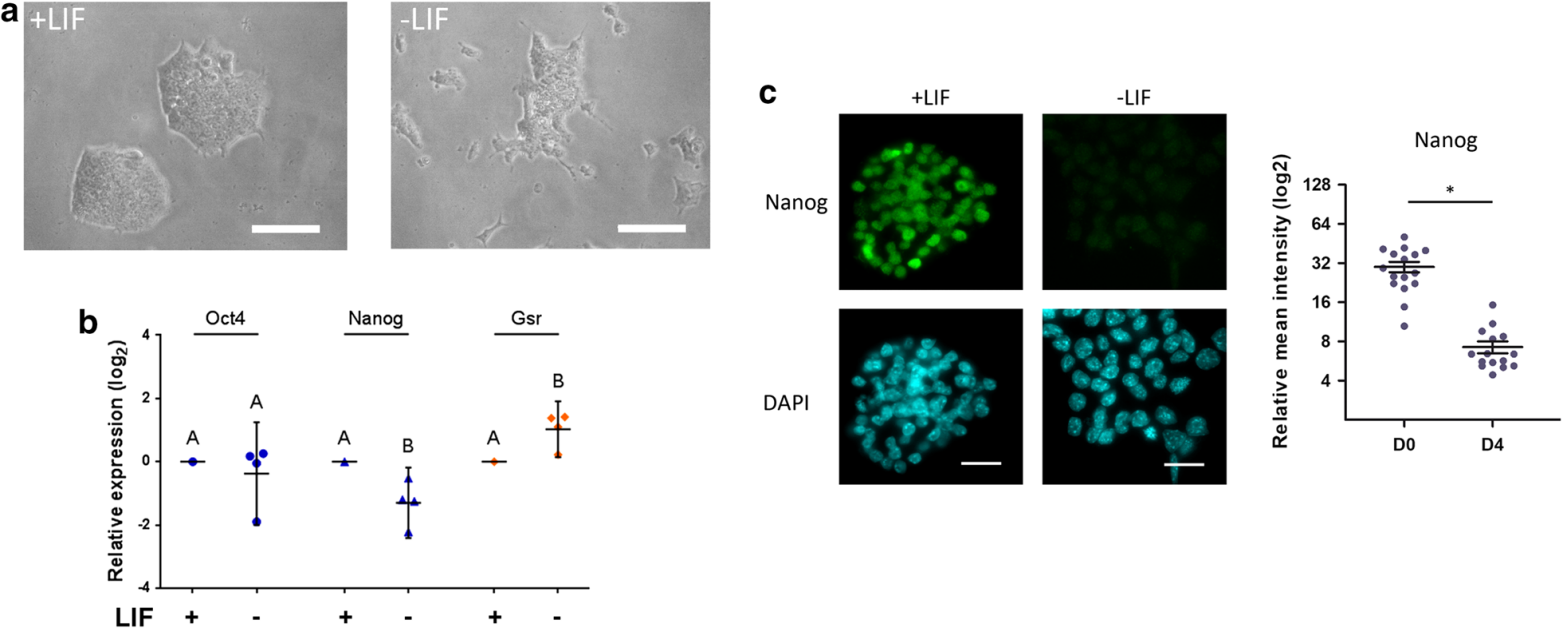
Gsr is upregulated in ESCs cultured without LIF. R1 ESCs were cultured under standard conditions in the presence of LIF, or in the absence of LIF for 4 days. **a** Representative pictures of cells cultured with LIF (+LIF) and without LIF (−LIF) after 4 days of treatment. Scale bars: 100 µm. **b** RNA was extracted and mRNA levels of Oct4, Nanog and Gsr were measured by RT-qPCR. Gene expression was normalized to the geometrical mean of Gapdh and Pgk1 expression and referred to the control condition (with LIF, shown as a line). Results are shown as mean ± SEM of four independent experiments (different letters indicate significant differences, p < 0.05). **c** Representative immunostaining of Nanog for R1 ESCs cultured in the presence of LIF (+LIF) or in the absence of LIF for 4 days (−LIF). Nuclei were counterstained with DAPI. Scale bars: 20 μm. The nuclear intensities were quantified and represented as dot plots, mean ± SEM are indicated, *p < 0.05

Based on the growing evidence that antioxidant defense system is modulated when pluripotent stem cells are induced to differentiate, and that glutathione reductase is an important enzyme involved in the cellular response to oxidative stress, we decided to study Gsr gene modulation. In a previous work, we have in silico analyzed the upstream region of the coding sequence of diverse genes involved in antioxidant system, including Gsr and found the presence of multiple predicted binding sites for the transcription factors Oct4 and Nanog in a 5 kbp region upstream the transcription start site of this gene [16]. In this work, we first studied Gsr gene modulation in undifferentiated ESCs and along differentiation. To this aim, we performed an in vitro differentiation protocol culturing R1 ESCs in standard stem cells culture medium supplemented with LIF, as control condition, and in absence of this cytokine for 4 days. We observed the expected change in cell morphology throughout the differentiation, as shown in Fig. 1a. Whereas ESC grew in compact and refringent colonies and showed high nucleus/cytoplasm ratio, differentiated cells grew as a monolayer and increased their cytoplasmic proportion. As shown in Fig. 1b, although Oct4 mRNA levels remained constant, Nanog mRNA levels diminished, confirming that cells left behind the undifferentiated state displaying the expected Nanog repression. The reduction in Nanog mRNA, quantified by RT-qPCR, reflects Nanog protein levels visualized by immunofluorescence (Fig. 1c). Notably, showing a reciprocal kinetics, when Nanog expression was reduced, Gsr mRNA levels were upregulated.

**Fig. 2:**
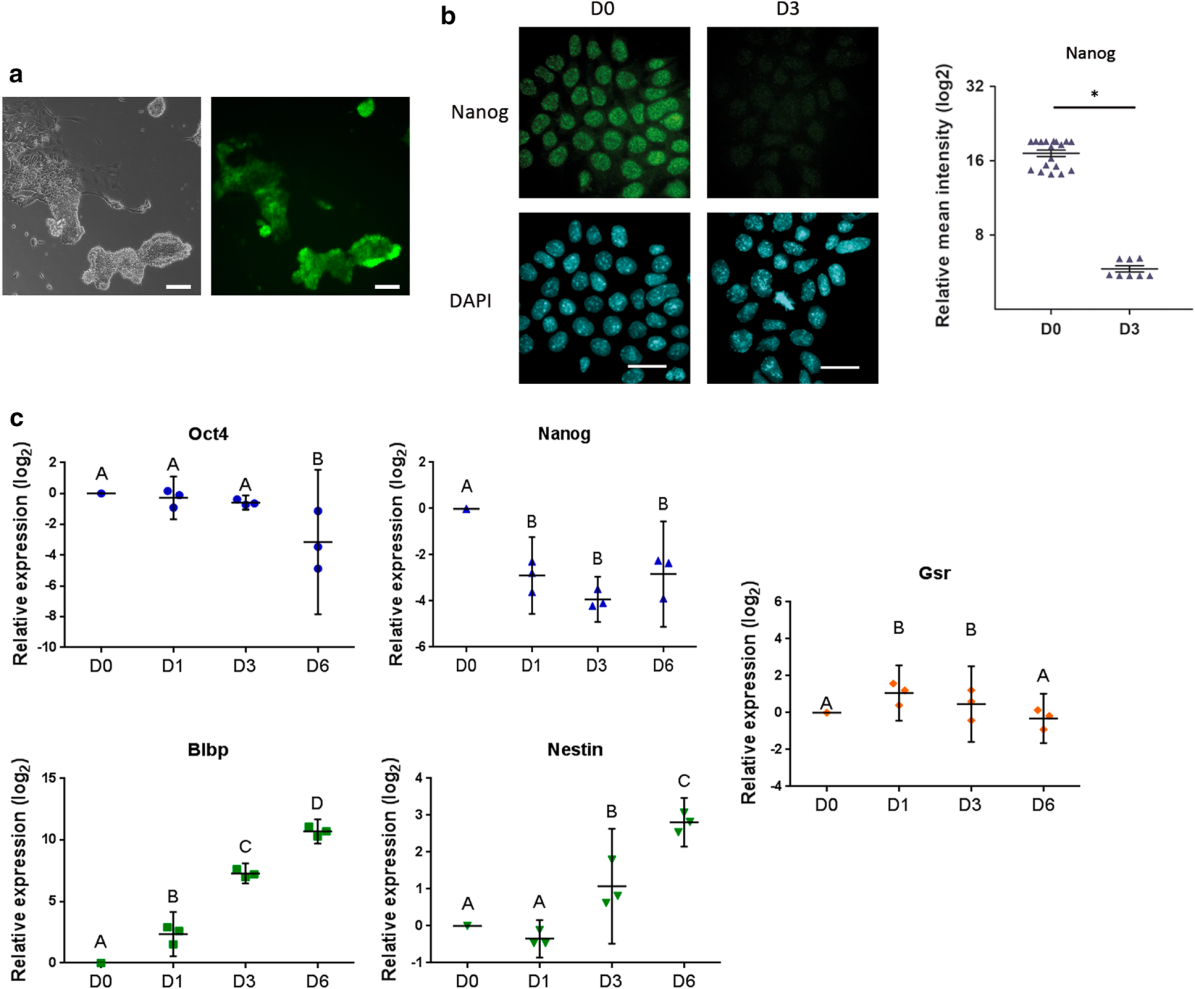
Gsr gene is modulated in ESCs along a neural progenitor differentiation protocol. 46C ESCs were subjected to a neural progenitor differentiation protocol. **a** Representative picture of cells at day 6 of differentiation showing expression of GFP, reporter of Sox1 promoter activity. Phase contrast, left panel; GFP, right panel. Scale bars: 100 µm. **b** Representative immunostaining of Nanog for 46C ESCs at days 0 (D0) and 3 (D3) of the neural progenitor differentiation protocol. Nuclei were counterstained with DAPI. Scale bars: 20 μm. The nuclear intensities were quantified and represented as dot plots, mean ± SEM are indicated, *p < 0.05. **c** RNA was extracted at days 0 (D0), 1 (D1), 3 (D3) and 6 (D6) after the induction of differentiation and mRNA levels of the indicated genes were measured by RT-qPCR. Gene expression was normalized to the geometrical mean of Gapdh and Pgk1 expression and referred to the control condition (D0). Results are shown as mean ± SEM of three independent experiments. Different letters indicate statistically significant differences between treatments (p < 0.05)

Next, to further investigate Gsr gene modulation we analyzed its expression pattern along a differentiation protocol to neural progenitor. We used the 46C ESC line for this approach, which is a reporter cell line that expresses GFP driven by Sox1 promoter, a specific marker of neuroectoderm [18]. We verified the success of the differentiation process by GFP fluorescence detection (Fig. 2a) and by the analysis of the expression of the pluripotency markers Oct4 and Nanog, and the neural markers Blbp and Nestin by RT-qPCR, at days 0, 1, 3 and 6, which behaved as expected (Fig. 2). In agreement with the previous result, we found that along with Nanog downregulation, visualized both at mRNA and protein levels (Fig. 2b, c), Gsr mRNA levels increased at days 1 and 3 of this neural precursor differentiation protocol.

#### Nanog transcription factor modulates Gsr gene expression

**Fig. 3:**
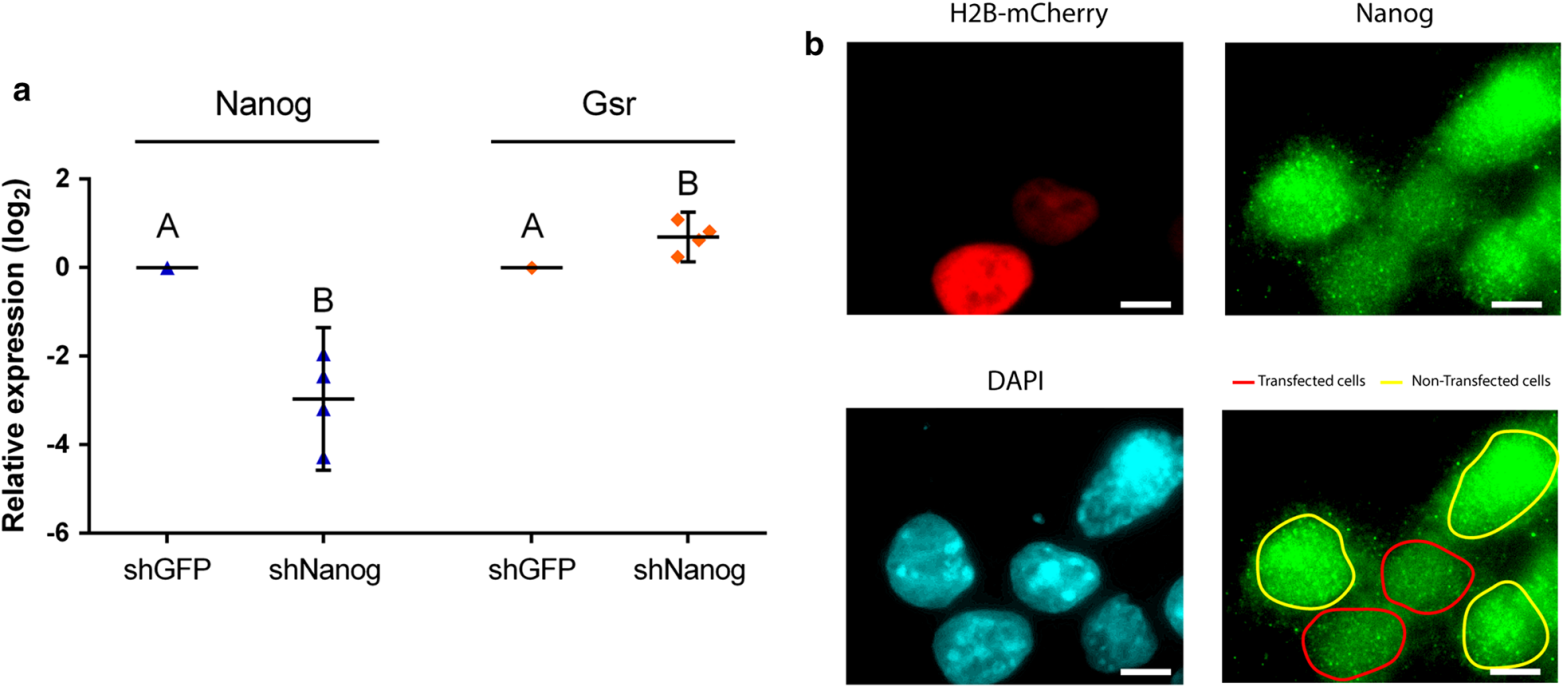
Gsr expression increases in ESCs transfected with a shRNA vector targeting Nanog. **a** R1 ESCs were transfected with pLKO.1-puro derived vectors targeting the transcription factor Nanog (shNanog) or eGFP (shGFP), as indicated under each bar. Then, transfected cells were selected with puromycin for 48 h and RNA was extracted. Nanog and Gsr mRNA levels were analyzed by RT-qPCR. Gene expression was normalized to the geometrical mean of Gapdh and Pgk1 expression and referred to the control condition (shGFP). Results are shown as mean ± SEM of four independent experiments. Different letters indicate statistically significant differences between treatments (p < 0.05). **b** R1 ESCs were co-transfected with shNanog and a vector encoding the fusion protein H2B-mCherry. 48 h after transfection, Nanog was visualized by immunofluorescence and nuclei were counterstained with DAPI. Transfected cells were identified by mCherry fluorescence detection. The figure corresponds to a representative image that shows that Nanog intensity signal is lower in transfected cells respect to non-transfected cells, evidencing Nanog downregulation by shNanog. Scale bars: 10 μm

Taking into account the induction of Gsr observed when Nanog was repressed along both differentiation protocols and the presence of six putative consensus sites for Nanog in the 3000 bp region upstream *Gsr* coding sequence, we decided to study whether Nanog modulates the endogenous *Gsr* gene expression by an shRNA approach. For this purpose, we downregulated this transcription factor’s mRNA levels using a shRNA targeting Nanog (shNanog). We transfected R1 ESCs with a vector encoding shNanog or targeting eGFP (shGFP) as control, and then analyzed Gsr mRNA levels by RT-qPCR. As outlined in Fig. 3, Nanog expression was downregulated by its specific shRNA, evidenced by RT-qPCR and immunofluorescence. Interestingly and according with our previous results, Gsr mRNA levels were greatly increased in Nanog-downregulated ESCs, suggesting a role in Gsr transcriptional regulation by this pluripotency transcription factor.

### Discussion

There is growing evidence showing that when pluripotent stem cells are induced to differentiate, antioxidant defense system is modulated [7, 10, 19, 20]. A key enzyme involved in the response to oxidative stress is glutathione reductase (Gsr), contributing to the preservation of this antioxidant molecule in precise levels. In a previous work, by in silico analysis, we found the presence of putative binding sites for the transcriptions factors Oct4 and Nanog in a 3000 bp region upstream Gsr coding sequence [16]. Considering this evidence and the hypothesis that ESCs’ specific transcription factors critical for pluripotency maintenance regulate genes differentially expressed along the differentiation process, we studied Gsr gene modulation. As mentioned before, regarding the differentiation process, it has been shown that ROS levels [7] and mitochondrial proliferation and activity increase during ESCs differentiation [9]. These reports propose that ROS are involved in the balance between self-renewal and differentiation. The antioxidant system is essential to maintain the adequate levels of these species and GSH is a key component for redox homeostasis [21]. The enzymes glutathione synthase and Gsr are responsible to keep GSH/GSSG at accurate levels. It was previously reported that Gsr gene expression decreased since day 7 of differentiation, in human pluripotent stem cells [7, 20]. On the other hand, in this work we found that Gsr mRNA levels were upregulated in mouse ESCs, at earlier time points of two distinct differentiation protocols. In accordance, it was reported that Gsr mRNA levels in E7 mouse embryos were high, and then they fall at later days reaching similar levels as in adult mouse [22].

As aforementioned, we found that Gsr gene was expressed in pluripotent stem cells and increased throughout differentiation, showing an expression pattern opposed to Nanog’s when these cells were induced to differentiate by two distinct protocols. We have previously found by in silico analysis, multiple putative binding sites for Nanog in Gsr promoter region [16]. Specifically, there are eight sequences similar to Nanog consensus preserving the AATG core sequence since positions − 523 to − 3612 from transcription start site (+ 1). Moreover, this transcription factor was found to be bound to the promoter region of Gsr in data from genome wide chromatin immunoprecipitation approaches in ESCs [23–27]. Furthermore, we used ChIP Atlas platform [28] to analyze ChIP-Seq experiments data and found evidences of functional Nanog regulatory regions in GSR genomic locus. The analysis revealed peaks indicating that Nanog was bound to Gsr gene, both upstream the transcription start site and in the first intron, in multiple experiments performed in ESCs (Additional file 1: Figure S1). In accordance to these results, we found an increase in Gsr mRNA levels when Nanog was downregulated using a specific shRNA targeting this transcription factor in ESCs. It was previously reported that Nanog represses at transcriptional level genes related to the differentiation process [29–31]. In this work, we found that this transcription factor modulates negatively Gsr expression, a gene involved in the antioxidant system. We have previously reported that Sod1 and Sod2, both genes from this system, are modulated by pluripotency transcription factors [16, 17]. As a whole, these results suggest that transcription factors essential for pluripotency maintenance such as Nanog, play a role in the homeostasis of redox status in ESCs.

### Conclusion

We found that Gsr, which is critical for maintaining GSH levels and cellular redox status, is modulated by the stemness transcription factor Nanog evidencing a link between pluripotency transcription factors and redox homeostasis. Deep understanding of the antioxidant system in pluripotent stem cells and the relationship between ROS and the differentiation process is crucial for future applications of these promising cells.

### Methods

#### Cell culture conditions and differentiation

R1 ESC line (ATCC) were cultured and differentiated as previously described [32–34]. 46C Sox1-GFP ESC line [18] (a kind gift from Austin Smith) was cultured and induced to differentiate to neural progenitor as previously described [16, 35, 36]. Cells were cultured until day 6 and efficacy of the differentiation protocol was analyzed by fluorescence microscopy.

#### Gene expression analysis

Gene expression was analyzed by RT-qPCR and immunofluorescence. A detailed description of the methodology, the antibodies and the sequence of the primers used is included in Additional file 2: Additional methods.

#### Nanog downregulation by shRNA transfection

R1 ESCs were transfected in p60 plates with 3 µg pLKO.1-puro derived vectors (Sigma), expressing shRNA targeting Nanog (shNanog, SHCLND-XM_132755) or eGFP (SHC005). Transfection, selection and mRNA analysis were carried out as previously described [16, 17]. For immunofluorescence, ESCs were co-transfected with shNanog and an expression vector encoding H2B-mCherry.

#### Statistics and data analysis

Results were presented as mean ± Standard error mean (SEM). Statistical comparisons were performed using Student’s *t* test, except for Fig. 2c, where data was analyzed by a linear mixed model and DGC Test was used for comparison between means. Residuals fitted normal distribution and homogeneity of variance; p values < 0.05 were considered significant. Analysis was performed with Infostat statistical software [37].

## Limitations

We attempted to evaluate Nanog effect on Gsr gene by ectopic expression in an heterologous system but we failed, presumably by the need of other factors missing in the system and/or different epigenetic landscape.

## Additional files

**Additional file 1: Figure S1.** Region of Gsr genomic locus including Nanog binding peaks according to Chip-seq experiments downloaded from the Chip-Atlas Database (https://chip-atlas.org/). Sequence Read Archive Database identifiers are indicated in the figure.

**Additional file 2.** Additional file contains Fig. S1 legend, Table S1 and additional methods.

## Data Availability

The dataset supporting the conclusions of this study and the information about the materials used is included within the article.

## Abbreviations

ESC: embryonic stem cell
Gapdh: glyceraldehyde-3-phosphate dehydrogenase
GSH: glutathione
Gsr: glutathione reductase
iPSC: induced pluripotent stem cell
Pgk1: phosphoglycerate kinase 1
ROS: reactive oxygen species
