# Supplementary figures and images for: The pluripotency transcription factor Nanog represses glutathione reductase gene expression in mouse embryonic stem cells
Source: BMC Res Notes. 2019 Jul 1;12:370. doi: 10.1186/s13104-019-4411-0 (PMC6604252; doi:10.1186/s13104-019-4411-0)

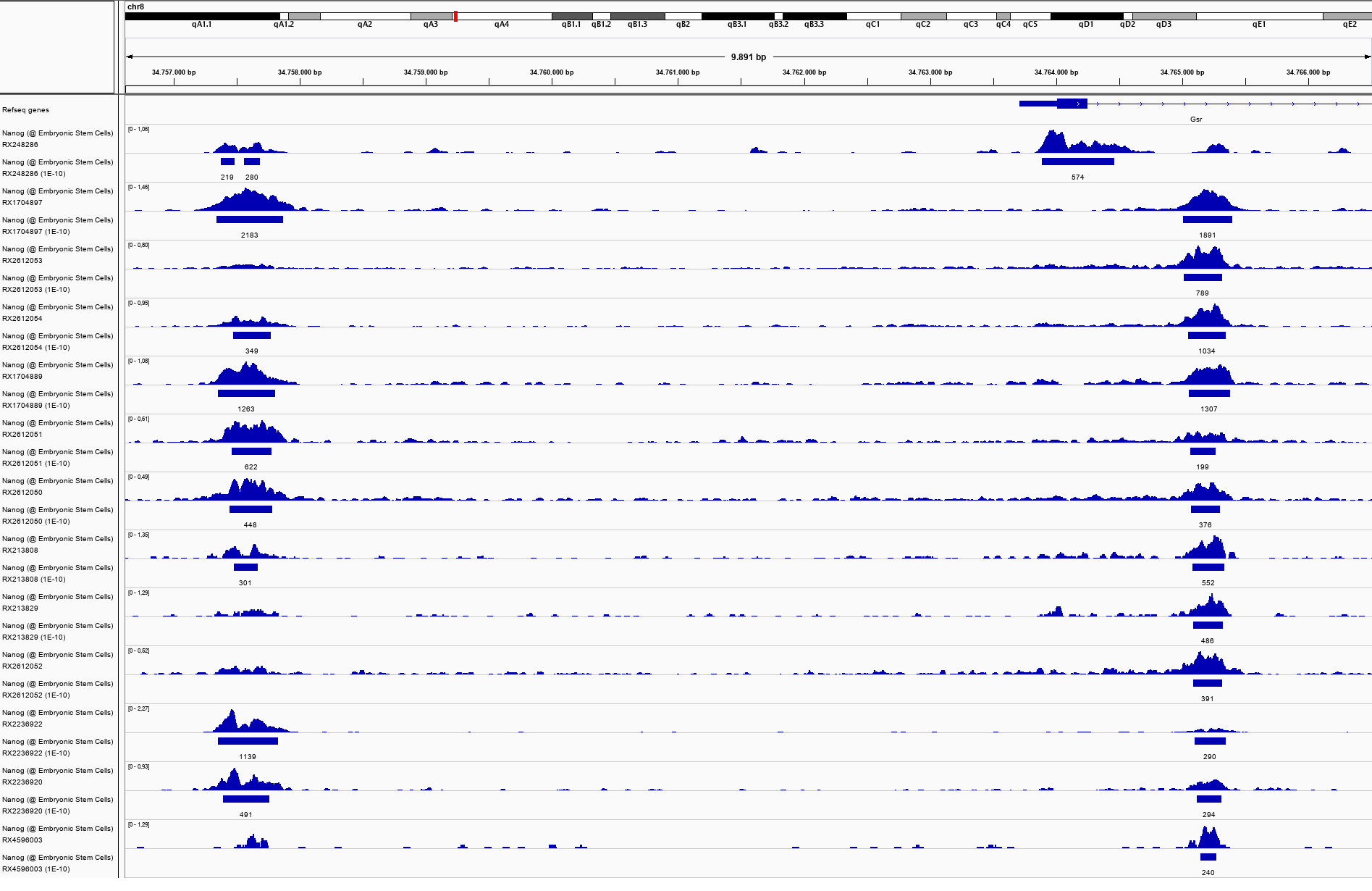

Supplement: Supplementary file 1 — Additional file 1: Figure S1. Region of Gsr genomic locus including Nanog binding peaks according to Chip-seq experiments downloaded from the Chip-Atlas Database (https://chip-atlas.org/). Sequence Read Archive Database identifiers are indicated in the figure. [file 13104_2019_4411_MOESM1_ESM.tif]
