# Supplementary material for: The pluripotency transcription factor Nanog represses glutathione reductase gene expression in mouse embryonic stem cells
Source: BMC Res Notes. 2019 Jul 1;12:370. doi: 10.1186/s13104-019-4411-0 (PMC6604252; doi:10.1186/s13104-019-4411-0)
Supplement: Supplementary file 2 — Additional file 2. Additional file contains Fig. S1 legend, Table S1 and additional methods. [file 13104_2019_4411_MOESM2_ESM.docx]

**Additional file**

**1. Additional Figure Legend**

**2. Additional Table**

**3. Additional Methods**

1. **Additional Figure Legend**

**Fig. S1.** Region of Gsr genomic locus including Nanog binding peaks according to Chip-seq experiments downloaded from the Chip-Atlas Database (<https://chip-atlas.org/>). Sequence Read Archive Database identifiers are indicated in the figure.

1. **Additional Table**

**Table S1. Primers used for RT-qPCR**

| Quantitative PCR primers | |  | |  |
| --- | --- | --- | --- | --- |
| Name | **Note** | | **Sequence (5’-3’)** | |
| Blbp | Forward | | GGGTAAGACCCGAGTTCCTC | |
|  | Reverse | | ATCACCACTTTGCCACCTTC | |
| Gapdh | Forward | | TGCCAAGGCTGTGGGCAAGG | |
|  | Reverse | | CGAAGGTGGAAGAGTGGG | |
| Gsr | Forward | | GGGTGGCACTTGCGTGAATG | |
|  | Reverse | | GGCGGCTCACATAGGCATCCC | |
| Nanog | Forward | | AGGGTCTGCTACTGAGATGCTCTG | |
|  | Reverse | | CAACCACTGGTTTTTCTGCCACCG | |
| Nestin | Forward | | CTGCAGGCCACTGAAAAGTT | |
|  | Reverse | | GACCCTGCTTCTCCTGCTC | |
| Oct4 | Forward | | TGACGGGAACAGAGGGAAAG | |
|  | Reverse | | TCAGCTTGGGCTAGAGAAGG | |
| Pgk1 | Forward | | TGGGCAAGGATGTTCTGTTC | |
|  | Reverse | | TGCAGTCCCAAAAGCATCAT | |

**Additional Methods**

**Quantitative RT- PCR**

Total RNA was extracted with Trizol (Thermo Fisher Scientific) following manufacturer’s instructions and reverse transcribed using MMLV reverse transcriptase (Thermo Scientific) and Random Primers (Invitrogen) according to the manufacturer’s instructions. Quantitative PCR amplification of DNA was performed using FastStart SYBR Green Master (Roche) and specific primers in a LightCycler 480 Real Time PCR system. Primers’ efficiency and N0 values were determined by LinReg software [10], and gene expression was normalized to GAPDH housekeeping gene N0, for each condition. Primers were designed using Primer3 software. Sequences for all primers used in RT-qPCR analysis are listed in the Supplemental Methods Tables section. All experiments were performed in at least three biological replicates, with at least two technical replicates for each condition.

**Immunostaining**

For immunofluorescence analysis, cells were grown on either gelatin coated coverslips or Lab-tek chamber slides (Nunc). Cells were fixed for 15 minutes with 4% paraformaldehyde, permeabilized with 0.1% Triton X-100 in PBS (PBST) and blocked with 3% normal donkey serum (Sigma) in PBST. Primary antibodies (Nanog: rabbit polyclonal, PEPROTECH 500-P236; mouse monoclonal, Santa Cruz Biotechnology sc-374001) in blocking solution were added to the samples that were incubated at 4°C overnight and washed three times in PBST for 30 minutes. The incubation with secondary antibodies (Donkey anti-Rabbit IgG Alexa Fluor 488, ThermoFisher A-21206; Donkey anti-Mouse IgG Alexa Fluor 488, ThermoFisher A-21202, respectively) and DAPI (Sigma) prepared in blocking solution was performed at room temperature for 30 minutes. Samples were washed as described before and imaged in an Olympus IX71 microscope.
